# The Ophthalmic Performance of Hydrogel Contact Lenses Loaded with Silicone Nanoparticles

**DOI:** 10.3390/polym12051128

**Published:** 2020-05-14

**Authors:** Nguyen-Phuong-Dung Tran, Ming-Chien Yang

**Affiliations:** Department of Materials Science and Engineering, National Taiwan University of Science and Technology, Taipei 10607, Taiwan; thaonguyeng89@gmail.com

**Keywords:** silicone nanoparticles, PDMS, TEOS, hydrogels, soft contact lenses

## Abstract

In this study, silicone nanoparticles (SiNPs) were prepared from polydimethylsiloxane (PDMS) and tetraethyl orthosilicate (TEOS) via the sol-gel process. The resultant SiNPs were characterized by dynamic light scattering (DLS), transmission electron microscope (TEM), and scanning electron microscope (SEM). These SiNPs were then blended with 2-hydroxyethylmethacrylate (HEMA) and 1-vinyl-2-pyrrolidinone (NVP) before polymerizing into hydrogel contact lenses. All hydrogels were subject to characterization, including equilibrium water content (EWC), contact angle, and oxygen permeability (Dk). The average diameter of SiNPs was 330 nm. The results indicated that, with the increase of SiNPs content, the oxygen permeability increased, while the EWC was affected insignificantly. The maximum oxygen permeability attained was 71 barrer for HEMA-NVP lens containing 1.2 wt% of SiNPs with an EWC of 73%. These results demonstrate that by loading a small amount of SiNPs, the Dk of conventional hydrogel lenses can be improved greatly. This approach would be a new method to produce oxygen-permeable contact lenses.

## 1. Introduction

Contact lenses are employed for correcting eye vision. The global market for contact lenses is about US$8.1 billion in 2018. Two major classes of soft contact lenses are silicone hydrogel and conventional hydrogel lenses. The market shares for the former is 69% while the latter takes 19% in 2018 [1]. Conventional hydrogel contact lenses are synthesized from hydrophilic monomers such as 2-hydroxyethyl methacrylate (HEMA), offering the wearer comfort due to their hydrophilicity. However, this class of contact lenses exhibits low oxygen permeability that may cause red-eye syndrome for long-term wearing [2,3]. To cope with this problem, silicon-containing polymers such as 3-(methacryloyloxy) propyltris(trimethylsiloxy) silane (TRIS) or polydimethylsiloxane (PDMS) are incorporated into the hydrogel to increase the oxygen permeability, leading to the inception of silicone hydrogel lenses [4,5,6,7,8,9,10,11].

Polydimethylsiloxane exhibits a hydrophobic nature and is low cost, simple to fabricate, and shows good biocompatibility, flexibility, thermal and oxidative stability, high optical transparency, and especially high oxygen permeability [12,13,14,15,16,17,18]. The main drawback of PDMS is the restriction of water absorptivity, surface wettability, and higher lipid deposition because of its inherent hydrophobicity [19,20,21]. These limitations can be improved through the combination with hydrophilic materials. The incorporation of hydrophobic and hydrophilic monomers usually takes two oppositional tendencies. The first trend is to increase equilibrium water content. However, this will decrease the oxygen permeability because of hydrophilic monomers. On the other hand, a higher PDMS content will enhance oxygen permeability but reduce the water uptake ability [11,22,23]. In silicone hydrogel contact lenses, water is a main factor to restrict the oxygen permeability [24,25,26]. However, the second trend may improve both wettability and oxygen transmissibility when hydrophobic and hydrophilic monomers are cooperated at a proper ratio.

In this work, a novel approach is adopted to improve the oxygen permeability of HEMA-based hydrogels. Instead of incorporating PDMS into the polymer chain, poly(dimethylsiloxane) dialkanol (PDMS diol) was reacted with tetraethyl orthosilicate (TEOS) through hydrolysis and condensation to form silicone nanoparticles (SiNPs) via the sol-gel process [27,28,29]. Thereafter, SiNPs were loaded into hydrogel synthesized from HEMA and 1-vinyl-2-pyrrolidinone (NVP). All the resultant hydrogels were subject to characterization, including Fourier transform infrared spectroscopy (FTIR), Raman, scanning electron microscope (SEM), transmission electron microscope (TEM), dynamic light scattering (DLS), equilibrium water content (EWC), oxygen permeability (Dk), optical transparency, mechanical strength, and contact angle measurements. We think this novel approach of loading silicone nanoparticles would improve the oxygen permeability without reducing the hydrophilicity and wettability of HEMA hydrogels.

## 2. Materials and Methods

### 2.1. Materials

Poly(dimethylsiloxane) dialkanol (PDMS-diol, KF-6001) was purchased from Shin-Etsu Chemical Co. Ltd., Tokyo, Japan. 1-Vinyl-2-pyrrolidinone (NVP), tetraethyl orthosilicate (TEOS), and 2-hydroxy-2-methylbenzene acetone (D-1173) were purchased from Sigma-Aldrich (St. Louis, Mo USA). Further, 2-hydroxyethylmethacrylate (HEMA) and ethylene glycol dimethacrylate (EGDMA) were obtained from Acros Organics (NJ, USA). Phosphate buffered saline solution (PBS, 0.1 M, pH 7.4) was prepared in our laboratory.

### 2.2. Preparation of Silicone Nanoparticles

**Figure 1:**
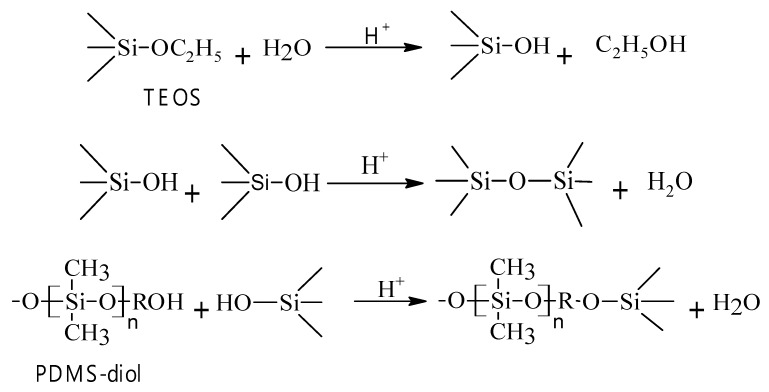
Preparation of silicone nanoparticles from TEOS and PDMS-diol.

Silicone nanoparticles (SiNPs) were synthesized by cross-linking PDMS-diol with TEOS through the sol-gel process shown in Figure 1. The reaction was conducted in a solution containing 0.1 mL of HCl (37%), 4 mL of water, and 2 mL of ethanol (95%). Subsequently, 1 mL of TEOS was added into the solution and then stirred for 1 h at room temperature, followed by adding 4 mL of PDMS-diol dropwise into the reacting solution. The reaction was stirred for 24 h at room temperature in the dark after completion of the addition. After condensing in a vacuum oven at 80 °C for 24 h, SiNPs were harvested and purified with ethanol before sonication and centrifugation. Finally, SiNPs were stored after drying in an oven at 80 °C for 12 h.

Figure 1 shows the synthetic reactions for silicone nanoparticles (SiNPs) from PDMS and TEOS. Firstly, the ethoxy groups of TEOS were hydrolyzed into the hydroxyl groups. In the subsequent condensation, silica and silicone were formed by removing hydroxyl groups of Si-OH [27,29,30].

### 2.3. Preparation of SiNPs-Loaded Hydrogel Composites

**Figure 2:**
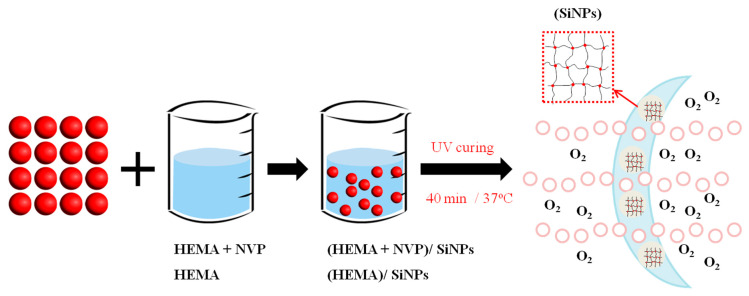
Preparation of SiNPs-loaded hydrogel lenses.

The hydrogels were polymerized from NVP and HEMA in the presence of SiNPs, cross-linking agent EGDMA and photo initiator D-1173 as shown in Table 1 and Figure 2. For all formulations, the mixture contained 0.5 wt% of EGDMA and D-1173. Then, the mixture was stirred in the dark at room temperature for 5 h. Afterward, the mixture was transferred to polypropylene molds and cured under UV light (365 nm) for 40 min. After demolding, lenses were soaked in 50% ethanol for 20 h at 50 °C to remove un-reacted monomers and photo initiator. Then, the lenses were immersed in distilled water for 4 h at 50 °C to wash out ethanol. Finally, the lenses were preserved in PBS (pH 7.4) at room temperature.

### 2.4. Elemental Analysis and Size of Particles

The elemental composition of dry particles was determined using a field emission scanning electron microscope (FE-SEM/EDS, JSM-6500F, JEOL, Tokyo, Japan). The particle size was analyzed using dynamic light scattering (DLS-DKSH, Malvern Instruments Ltd., Malvern, UK), and transmission electron microscopy (TEM, JEM-2000FXII, JEOL, Japan).

### 2.5. Equilibrium Water Content

The equilibrium water content (EWC) of the hydrogel was calculated as follows:

(1) $$\mathrm{EWC}\;(\%)=\frac{W_{2}-W_{1}}{W_{2}}\times 100$$

where W_1_ and W_2_ are the weights of the dry lens and the rehydrated lens in distilled water for one day at room temperature, respectively.

### 2.6. Optical Transparency

After swelling in PBS solution, the lens was adhered on the surface of cuvette containing 2 mL distilled water. The optical transparency was determined in a wavelength range of 400–700 nm using a UV-Vis spectrophotometer (Cary 300, Agilient Technologies, Santa Clara, CA, USA).

### 2.7. Surface Characterization

The contact angle of the contact lens was measured using a contact angle goniometer (DSA 100, Krüss GmbH, Hamburg, Germany) at room temperature. The contact angle was an average of three repetitions.

### 2.8. Chemical structure

The structure of SiNPs and contact lenses were examined using Raman Spectroscopy and FTIR. The FTIR of SiNPs (Nicolet 170 SX, Thermo Fisher Scientific, Madison, WI, USA) were performed in the wavenumber range of 600–4000 cm^−1^. SiNPs were pelletized with potassium bromide (KBr) before being scanned over 32 times by an infrared ray. The lenses were detected over 32 scans based on FTIR-ATR. The Raman spectroscopy (iHR550, Horiba Scientific, Kyoto, Japan) was determined in the wavenumber range of 400–4000 cm^−1^.

### 2.9. Mechanical Properties

The mechanical properties of hydrogel specimens were determined by modulus and tensile strength. Samples were cut as dog bone shape after hydrated in DI water. Modulus and tensile strength of specimens were measured based on a tensile tester (MTS 810, Material Test System, Eden Prairie, MN, USA) via ASTM D1708 standard at a crosshead speed of 50 mm/min.

### 2.10. Oxygen Permeability

The oxygen permeability (Dk, barrer) of the lens was determined according to ISO18369-4:2006 which is based on polarographic method using an oxygen permeometer (201 T O2 permeometer, Createch, Chesterfield Twp, MI, USA). Polarography measures the oxygen permeation through a sample by measuring the current produced in a cell by reducing oxygen at a noble metal electrode. Before testing, guard ring polarographic cell (8.6 mm radius, CreaTech/Rehder Development Co., Chesterfield Twp, MI, USA), buffer solutions and the lenses were placed in a temperature and humidity-controlled box at 37 °C and 98% relative humidity till the temperature equilibrium. After fully hydrated, the lenses were stacked to measure the electronic current at a various number of lenses to correct the boundary effect. The linear plot of t/Dk versus thickness was drawn and determined Dk/t from the slope [31,32,33].

## 3. Results and Discussion

### 3.1. Size and Elemental Composition of Particles

**Figure 3:**
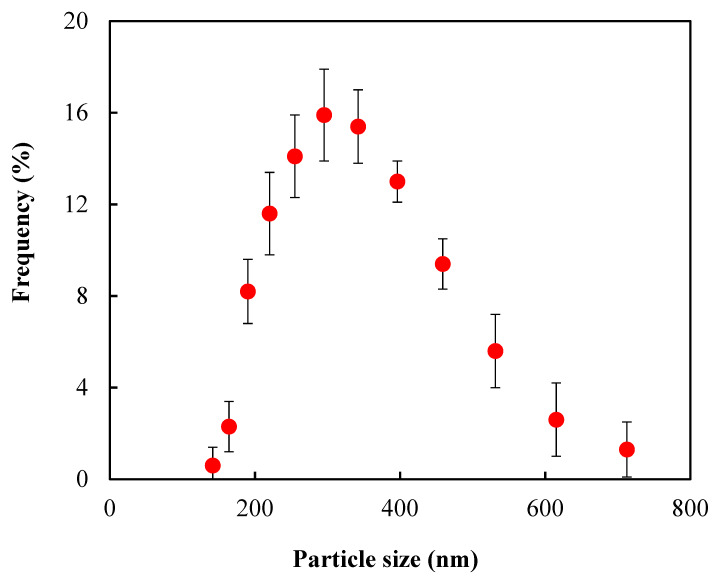
Size distribution of silicone nanoparticles obtained from DLS.

**Figure 4:**
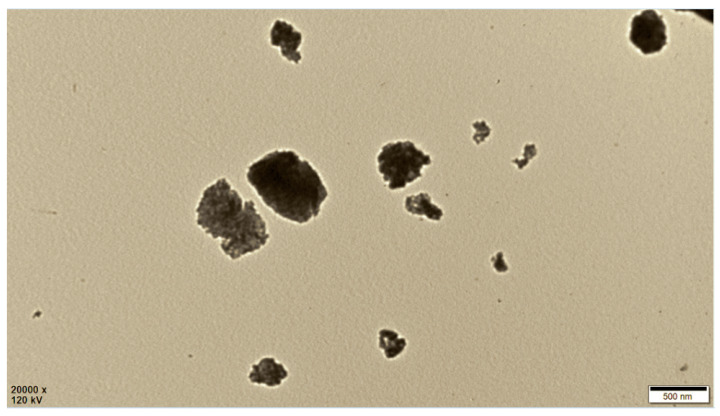
TEM image of SiNPs.

The size of SiNPs was determined based on DLS measurement as shown in Figure 3. The average diameter of SiNPs was 330 ± 100 nm. The TEM image in Figure 4 shows similar SiNPs sizes.

**Figure 5:**
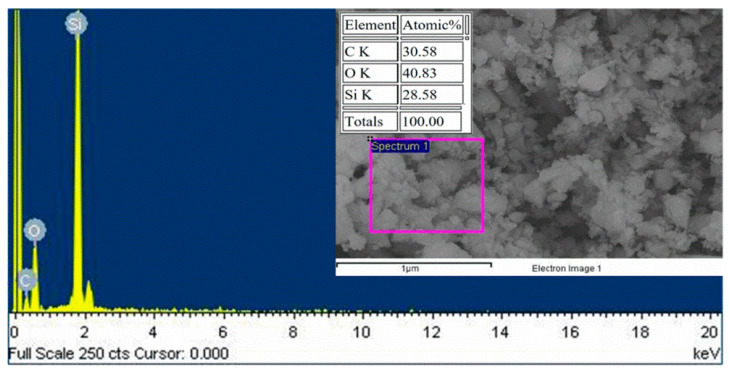
Micrograph of SiNPs from SEM-EDS.

The hydrolysis and condensation reaction of TEOS would produce silica particles based on the sol-gel process [27,28,29,34]. In this work, the condensation of TEOS occurred in the presence of PDMS-diol to result in silicone nanoparticles. The EDX results in Figure 5 showed that the elemental composition of SiNPs was consisted of 28.6% Si, 40.8% O, and 30.6% C. Considering that the atomic ratio of Si:O in PDMS is 1:1 while that in SiO_2_ is 1:2, the mole fractions of PDMS and SiO_2_ in SiNP can be estimated by solving the following equations

$$\begin{array}{l} \left\{ \begin{array}{ll} x+y=0.286\; & \mathrm{for}\;\mathrm{Si}\\ x+2y=0.408 & \mathrm{for}\;O \end{array}\right.\\ ∴\;y=0.122,\;x=0.164 \end{array}$$

Thus, the mole fraction of silicone is $\frac{x}{x+y}=\frac{0.164}{0.286}$ = 57.4% and that of silica is $\frac{y}{x+y}$ = 42.6%. In addition, the presence of carbon in SiNPs indicated that PDMS did incorporated into SiNPs.

**Figure 6:**
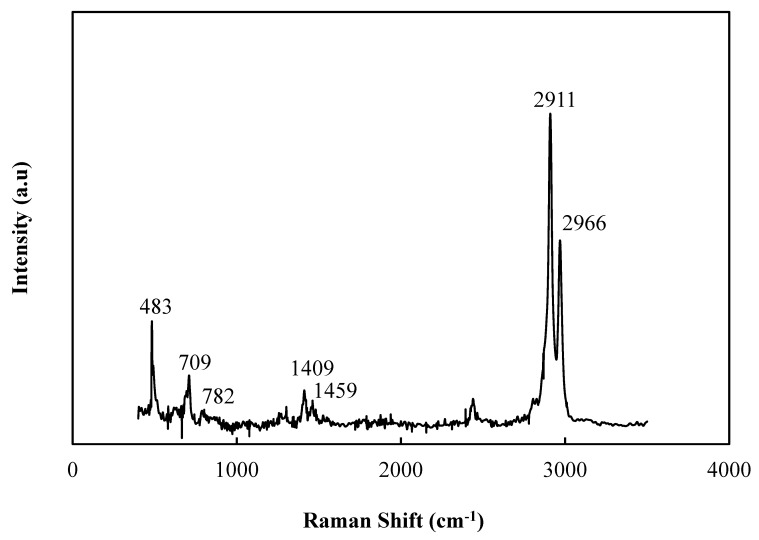
Raman spectrum of SiNPs.

**Figure 7:**
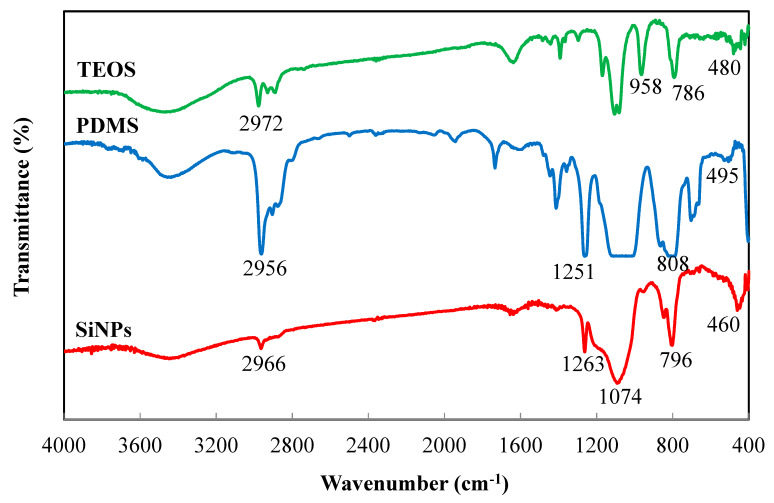
FTIR spectra of TEOS, PDMS, and SiNPs.

Raman and FTIR were employed to examine the chemical structure of SiNPs synthesized from PDMS and TEOS. Figure 6 shows that the Raman spectrum of SiNPs exhibited strong PDMSs peaks appearing at 2909–2968 cm^−1^, 1409–1459 cm^−1^ (C-H groups), and 706–782 cm^−1^ (Si-C groups). These results were also found in the literature [35,36,37]. Furthermore, the peak at 483 cm^−1^ were attributed to Si-O-Si [38]. These characteristic peaks indicated that the reaction of PDMS and TEOS through the sol-gel process was successful. Comparing the spectra of TEOS, PDMS, and SiNPs in Figure 7, the peaks of Si-O-Si (1074 cm^−1^, 808 cm^−1^, 796 cm^−1^, 786 cm^−1^, 495 cm^−1^, 480 cm^−1^, 460 cm^−1^) and SiOH (958 cm^−1^) were observed. The stretching vibrations of C-H occurred at 2792 cm^−1^ of TEOS, 2956 cm^−1^ of PDMS, and 2966 cm^−1^ of SiNPs while the peaks of Si-C (1251 cm^−1^ and 1263 cm^−1^) were respectively observed in the spectra of PDMS and SiNPs [24].

### 3.2. Optical Transparency

**Figure 8:**
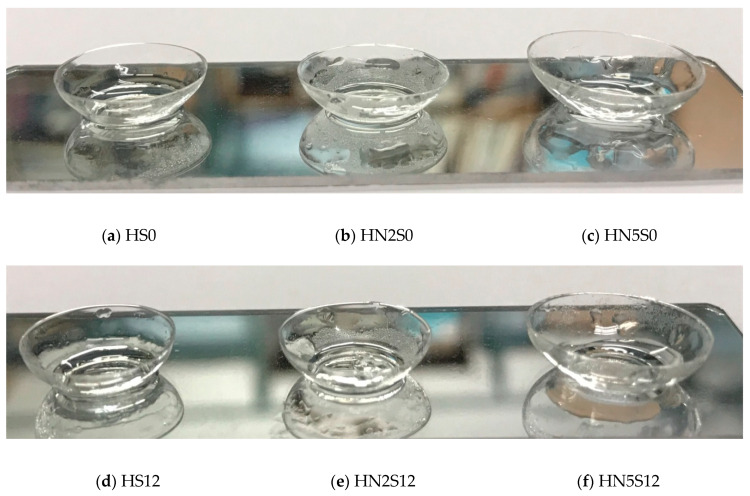
Photos of hydrated contact lenses.

Figure 8 shows the photos of hydrated soft lenses including poly(HEMA-co-NVP) and poly(HEMA-co-NVP)-SiNPs. These lenses appeared transparent.

**Figure 9:**
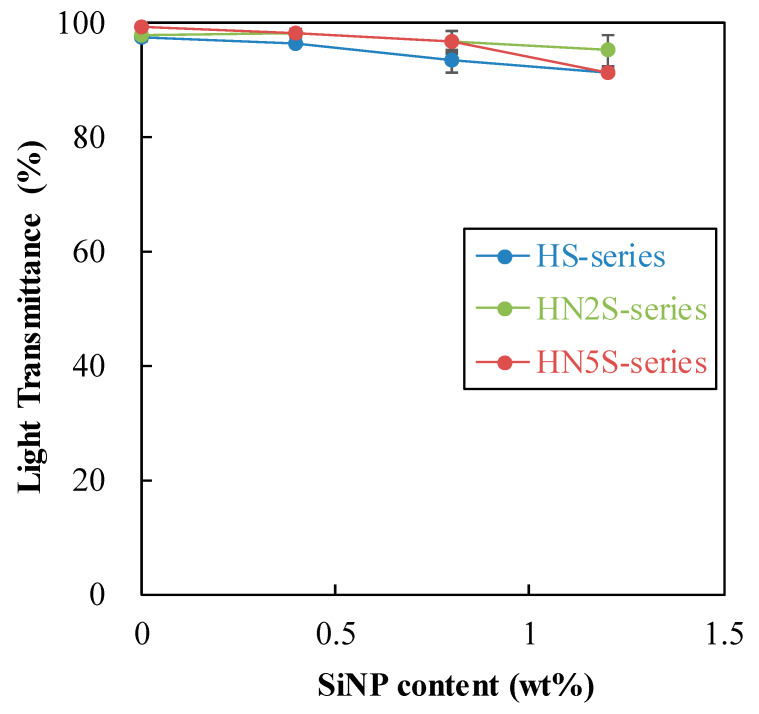
Light transmittance of SiNPs samples.

Figure 9 shows that the light transmittance (T%) of the contact lens decreased with the increase of SiNPs content. The reduction in transparency can be attributed to the size distribution of SiNPs (see Figure 4) that caused light scattering. At a content of 1.2 wt%, the transmittance dropped to 90%. The light transmittance of a contact lens is preferred to be above 90% [39]. A higher SiNPs content would further decrease the transparency of the contact lens below 90%. Thus, in this study, the maximum content of SiNPs was limited to 1.2 wt%.

### 3.3. Equilibrium Water Content

Equilibrium water content (EWC) is an important index conferring comfortable wearing for patients because of the softness and wettability as well as the limitation of dry corneal eye [40,41]. In this present work, three series of soft lenses were prepared with different EWC values. The basic ingredient of these hydrogels was HEMA, which is a well-known monomer for contact lens. The other ingredient, NVP, is to increase the hydrophilicity of the hydrogel. All these formulations were crosslinked with 0.5 wt% of EGDMA.

**Figure 10:**
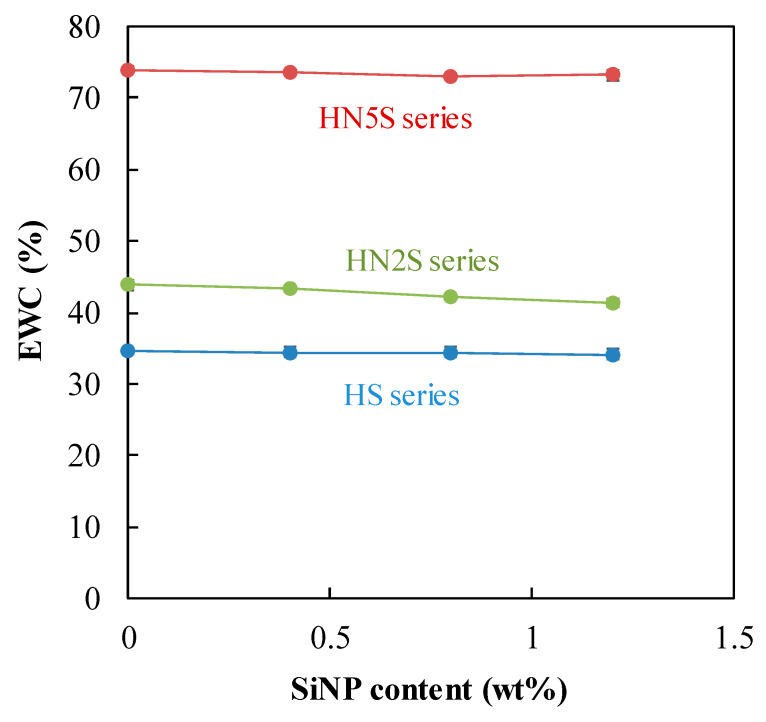
EWC of SiNPs samples.

Figure 10 shows that the presence of SiNPs in hydrogel network did not significantly affect the EWC of soft lenses. The values of EWC for the HS-, HN2-, and HN5- series varied around 34%, 42%, and 73%, respectively, regardless of the content of SiNPs. This is because that the content of SiNPs was low, and that these nanoparticles interacted little with the matrix of HEMA and NVP. In other words, a small number of nanoparticles were simply dispersed in the hydrogel matrix. On the contrary, for commercialized silicone soft lenses, hydrophobic ingredients such as TRIS, SiMA, and PDMS were incorporated into the main chains of the hydrogel and caused the reduction in EWC of contact lenses [19,22,42,43].

### 3.4. Contact Angle

**Figure 11:**
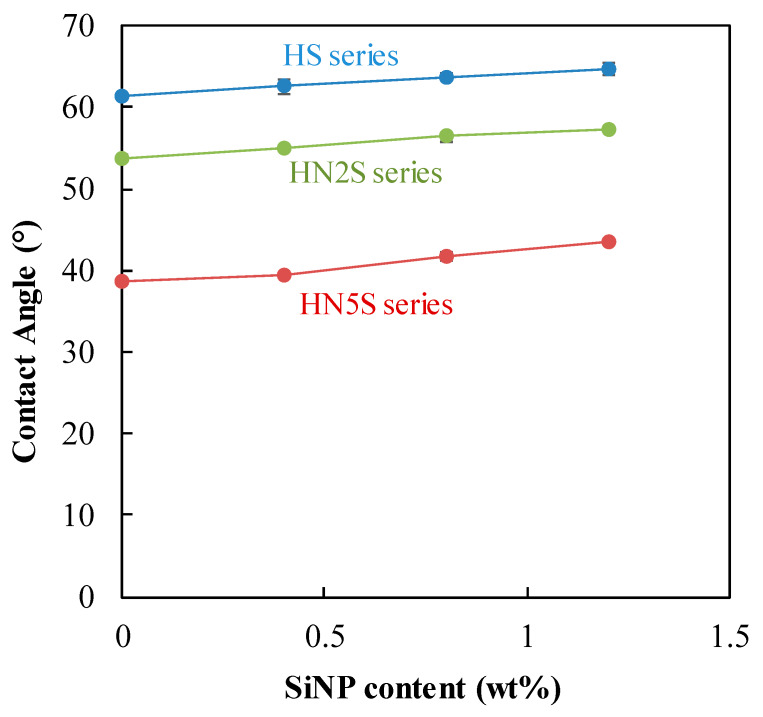
Contact angle of SiNPs samples.

Figure 11 shows that the contact angle of the soft lenses increased slightly with the content of SiNPs. This may be attributed to the inherent hydrophobic PDMS in SiNPs embedded in hydrogel matrix [24,44,45]. However, the difference was less than 5°, and all the contact angles were below 70°. Thus, the addition of SiNPs did little to change the wettability of these lenses. The slight increase may be attributed to a small quantity of hydrophobic PDMS (from SiNPs) exposed on the surface [23].

### 3.5. IR Spectra of Contact Lenses

**Figure 12:**
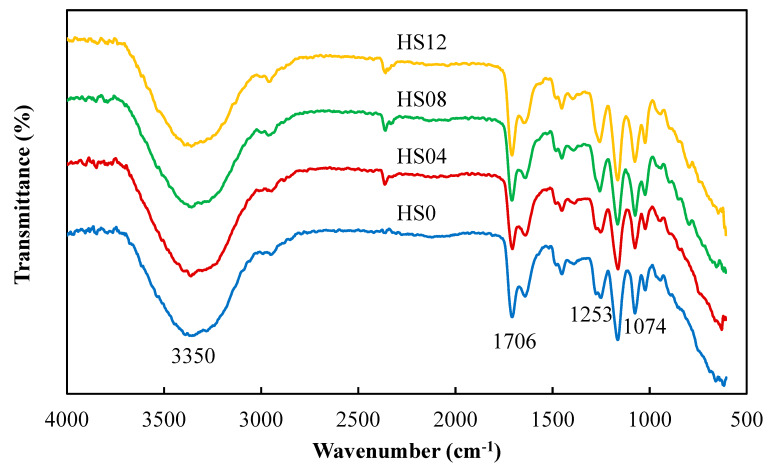
The FTIR spectra of HS- series SiNPs-containing contact lenses.

Figure 12 shows that the FTIR spectra of HS-series lenses differed little as the contents of SiNPs increased from 0 wt% to 1.2 wt%. This phenomenon was observed for HN2S- and HN5S- series as well (not shown). In the spectra of HS-series lenses, peaks of OH groups appeared at 3350 cm^−1^ while peaks of Si-O-Si groups were detected at 1074 cm^−1^. Further, in the spectrum of HS-series, the presence of Si-C and C=O were respectively observed at the peaks of 1253 cm^−1^ and 1706 cm^−1^ [24]. In the spectra of HN2S- and HN5S- series, the peaks of OH (3338, 3338 cm^−1^) and Si-O-Si (1072, 1080 cm^−1^), Si-C (1257 cm^−1^, 1257 cm^−1^) and C=O (1643 cm^−1^, 1631 cm^−1^) were also detected.

### 3.6. Mechanical Properties

**Figure 13:**
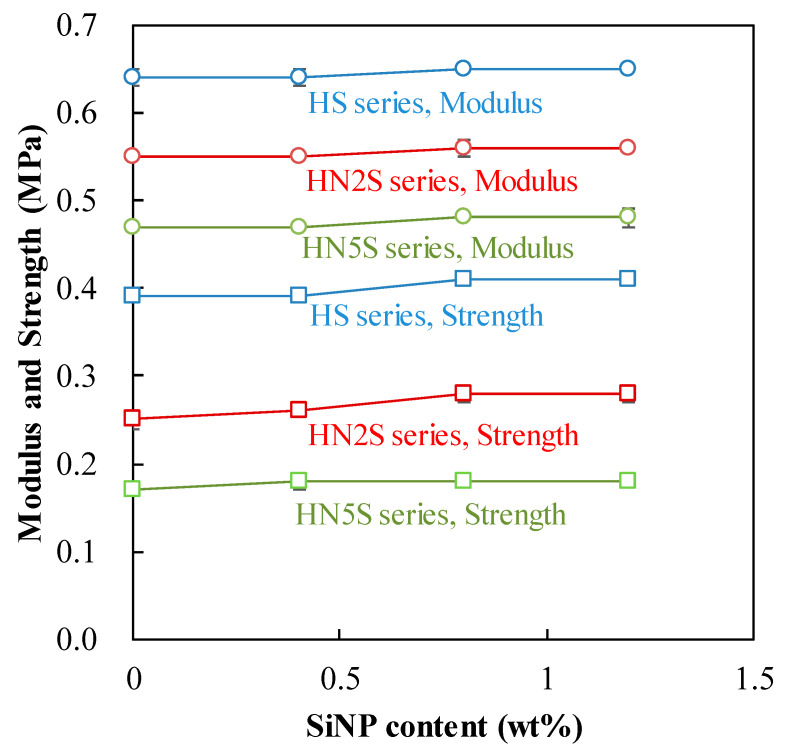
The modulus and strength of SiNPs samples.

Figure 13 presents the modulus and tensile strength of all HS, HN2S, and HN5S series. The moduli of all HN5S samples were not significantly affected by the SiNPs content increasing from 0 wt% to 1.2 wt%. Particularly, the moduli of all HN5S lenses were approximately 0.48 MPa for all SiNPs contents. This tendency was also found for HS and HN2S series samples. The moduli of HS and HN2S samples were approximately 0.65 MPa and 0.56 MPa, respectively. In this research, the moduli of all HN5S, HN2S, and HS series were around 0.48–0.65 MPa, which were similar to commercial lenses such as Acuvue Oasys, Acuvue Advance, and Biomedics 38 [43,46]. Hence, these SiNPs-contained contact lenses exhibited mechanical properties comparable to those commercial contact lenses.

### 3.7. Oxygen Permeability

**Figure 14:**
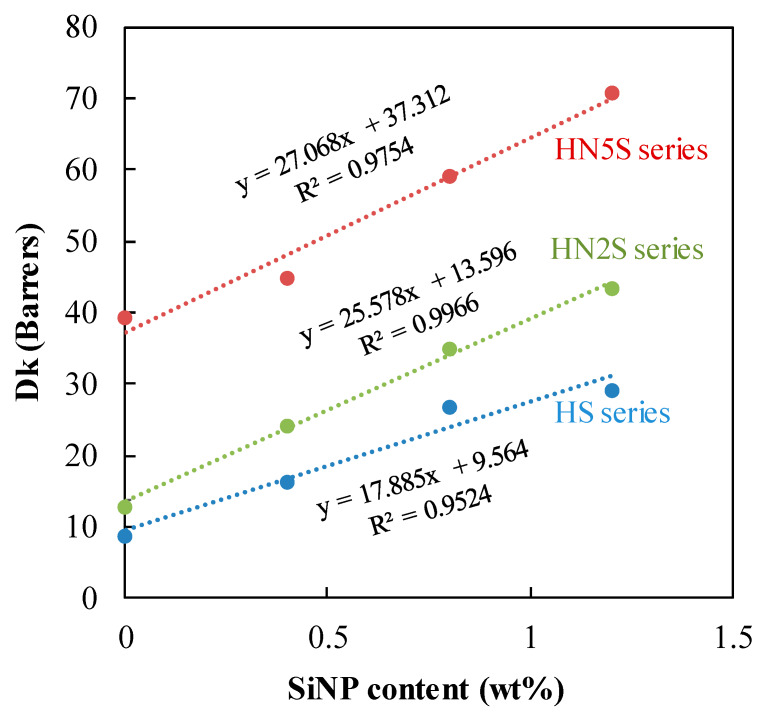
The effect of SiNPs content on Dk.

Figure 14 shows that Dk is linearly depending on the SiNPs content. Furthermore, the slop increased with the hydrophilicity. Although the Dk increases with the EWC for conventional non-silicone hydrogels, the loading of SiNPs did accelerate the permeation of oxygen in these composite hydrogels. For the HS series, the Dk increased from 9 to 29 barrer as the content of SiNPs increased to 1.2 wt%. For a more hydrophilic series, Dk of HN5 soft lenses increased rapidly from 39 to 71 barrer when the content of SiNPs increased from 0 to 1.2 wt%. Additionally, all formulations containing a higher concentration of NVP monomers exhibited higher oxygen transmissibility than others. As a result, the oxygen permeation of three formulation series including p(HEMA-*co*-NVP) and pure HEMA polymers was affected by the SiNPs content.

It is well-known that hydrophobic PDMS can improve oxygen transmissibility of soft lenses based on its siloxane groups (-Si(CH_3_)_2_-O-), especially silicon-oxygen bond [7,9,47]. However, the main weakness of the hydrophobic component is to impair the water absorbability of the lens, [16,24,48] thus EWC and Dk usually follow an inverse correlation. Accordingly, water is usually the limiting factor of oxygen transport for silicone hydrogel lenses [26,49]. On the contrary, for the SiNPs-loaded hydrogel, the addition of SiNPs did not affect the water uptake ability while increasing the oxygen permeability of lenses, as shown in Table 1. This phenomenon broke the reverse relationship of EWC and Dk in comparison to other non SiNPs lenses.

For hydrogels loaded with SiNPs, the content of these nanoparticles was less than 1.2 wt% of the matrix. Although SiNPs were hydrophobic, the effect on the water absorbability was low due to this small content. Furthermore, hydrophilic matrix of the hydrogel interacted little with these hydrophobic nanoparticles. Thus, the mobility of these nanoparticles would be higher than in the case of silicone hydrogels where PDMS chains were integrated into the matrix. Apparently, higher mobility would facilitate the permeation of oxygen through the lens. Although in this study the highest Dk was 70 barrer, we believe that higher Dk could be attained for higher SiNPs content once the particle size was reduced through improving the synthesizing process of SiNPs, thereby breaking through the transparency limitation.

## 4. Conclusions

As demonstrated in this study, silicone nanoparticles (SiNPs) were synthesized from TEOS and PDMS-diol. The existence of PDMS and TEOS in SiNPs was verified through Raman and FTIR spectroscopy. The resultant nanoparticles exhibited a diameter of 330 ± 100 nm and composed of 57% of silicone and 43% of silica. These nanoparticles were further entrapped in hydrogels polymerized from HEMA and NVP. The resultant SiNPs-loaded hydrogel lenses exhibited an unusual correlation between the oxygen permeability (Dk) and the equilibrium water content (EWC): the Dk increased with the content of silicone nanoparticles while the EWC changed insignificantly. Moreover, based on the result of the contact angle and Young’s modulus, the loading of SiNPs slightly influenced the wetting surface and mechanical properties. The transparency was reduced to 91% when the content of SiNPs was 1.2 wt%, probably due to the light scattering from the nanoparticles. Further effort to reduce the particle size is underway in our lab. With this work, we demonstrate a novel approach to improve the oxygen permeability without impairing the hydrophilicity of soft contact lenses. These results would be beneficial to the development of soft contact lenses.

## Figures and Tables

**Table 1:** Formulation of soft lenses including HEMA, NVP, and silicone nanoparticles (SiNPs).

Sample	Gel Composition (mg)		
	HEMA	NVP	SiNPs
HS0	100	0	0
HS04	100	0	0.4
HS08	100	0	0.8
HS12	100	0	1.2
HN2S0	80	20	0
HN2S04	80	20	0.4
HN2S08	80	20	0.8
HN2S12	80	20	1.2
HN5S0	50	50	0
HN5S04	50	50	0.4
HN5S08	50	50	0.8
HN5S12	50	50	1.2
